# The education gap over immigration and socioeconomic security

**DOI:** 10.1111/1468-4446.12959

**Published:** 2022-06-25

**Authors:** Matthijs Rooduijn

**Affiliations:** ^1^ Department of Political Science University of Amsterdam Amsterdam The Netherlands

**Keywords:** education, immigration, polarization, public opinion, social spending

## Abstract

Worries about polarization are on the rise. In today's Europe, one of the most manifest gaps is the education divide over immigration. Where lower educated citizens tend to be negative about immigration, higher educated individuals are generally positive. Yet the magnitude of this education divide strongly differs between countries. What explains these differences? I theorize that when the levels of socioeconomic security are high, in particular less well educated citizens will be more likely to focus on issues with a strong cultural component, like immigration, and therefore hold more radical opinions. As a result, existing divides will be more pronounced. Analyzing 23 countries between 2002 and 2018, I show that social welfare spending fuels the education divide over immigration. I demonstrate that, indeed, it does so by affecting the immigration attitudes of the less well educated—not those of the better educated.

## 1 INTRODUCTION

In Europe and the United States discussions about immigration have fueled intense debates, which have led to new political divides. The newspaper *The Economist*, for instance, proclaimed that “the political divide that matters is less and less between left and right, and more and more between open and closed”.^1^ In today's Europe, this divide most clearly expresses itself along the lines of education (Bovens & Wille, 2017): where lower and middle educated citizens tend to be more nationalist, higher educated citizens are generally more cosmopolitan (Citrin et al., 1997; Hainmueller & Hiscox, 2007; Kunst, 2022). Yet these education gaps strongly differ between countries (Coenders & Scheepers, 2003; Finseraas, 2019; Hello et al., 2002; Weil, 1985). How can these differences be explained?

My main argument is that economic security plays an important role. I propose that when levels of social spending are high, and citizens have good reasons to feel more economically secure, in particular less well educated citizens (who are generally more socioeconomically vulnerable than their higher educated counterparts) will be more likely to focus on issues with a strong cultural component, like immigration. As a result, when it comes to such issues, the existing differences between education groups can be expected to be more pronounced.

Based on an analysis of individual‐level data coming from the European Social Survey (ESS, 2002–2018), and aggregate‐level information from various other sources, I show that, indeed, welfare state generosity fuels the education divide over immigration. And, as expected, the source of this divide is that social spending affects in particular the immigration attitudes of the less well educated. These findings are highly relevant for current debates about social polarization, because they show that welfare state generosity can have negative sociopolitical consequences.

## 2 EDUCATION DIVIDES OVER IMMIGRATION

Western societies are divided over sociocultural issues. Kriesi et al. (2006, 2008) have shown that the “winners of globalization”—those with higher socioeconomic positions who profit from international competition—tend to hold more positive attitudes toward issues like immigration and European unification, whereas the “losers of globalization”—those with lower socioeconomic positions who feel threatened by the opening of borders—are likely to be more negative about these issues.

This division between “winners” and “losers” can be expected to be especially outspoken along educational lines. Bovens and Wille (2017) argue that a person's educational background has become an increasingly important predictor for his or her attitudes and political behavior. In general, lower and medium educated citizens tend to hold more nationalist anti‐immigration attitudes, whereas the higher educated are more cosmopolitan and pro‐immigration (Citrin et al., 1997; Hainmueller & Hiscox, 2007; Kunst, 2022).

There exist various explanations for this education effect. First, it could be that higher educated citizens are less likely to feel threatened by immigrants than lower educated individuals, because they generally have higher skill levels than immigrants and therefore do not experience competition (Scheepers et al., 2002). Second, it could be the case that those with a higher education level are more positive about immigration because the education system fosters cosmopolitan and tolerant attitudes (Stubager, 2010). A third possibility is that attitudinal differences already exist before someone starts his or her secondary education (Lancee & Sarrasin, 2015). Yet whatever the exact causal story, education seems to be strongly related to immigration attitudes.

Previous studies have shown that the education gap vis‐à‐vis immigration attitudes is virtually universal in that it exists in basically every country (Hjerm, 2001). At the same time, however, the magnitude of the divide strongly differs between countries (Coenders & Scheepers, 2003; Finseraas, 2019; Weil, 1985). What explains these differences?

## 3 SOCIOECONOMIC CONDITIONS

Kriesi et al. (2008, p. 28) argue that the divide between “winners” and “losers” of globalization can be expected to be particularly strong when the economy performs well (see also Kalmijn & Kraaykamp, 2007). When the levels of economic security are high, people will “give increasing emphasis to other types of needs” (Inglehart, 1977, p. 22). In other words, if the macro‐level circumstances of a polity at a certain point in time foster economic security, they will likely make people shift their focus from purely economic issues to issues with a strong cultural component, like immigration.^2^ Under such conditions it can be expected that in particular the lower and medium educated will feel more secure. After all, these education groups can be expected to be more vulnerable to economic risks than the higher educated. Hence, if citizens have good reasons to feel economically secure, the existing cultural differences between education groups can be expected to be more pronounced.

## 4 RESEARCH DESIGN

I make use of individual‐level data provided by the European Social Survey (ESS) (2002–2018) and aggregate‐level data coming from various other sources. Data were available for 23 countries. The dependent variable in my analysis is *immigration attitudes* ranging from “0” (anti‐immigration) to “10” (pro‐immigration). The main independent variable, *education*, is a dichotomous variable based on the ES‐ISCED categorization provided by the ESS (0 = “lower and medium educated”; 1 = “higher educated”). The reason I recoded this variable like this is that Bovens and Wille (2017) have convincingly shown that the education divide is particularly pronounced if one compares the higher educated on the one hand with the other categories on the other.^3^ To make sure that my assessment of the education divide over immigration is not confounded by other variables, I controlled for various individual‐level characteristics. See for detailed information about all variables in the Sections A and B in Supporting Information S1.

**FIGURE 1:**
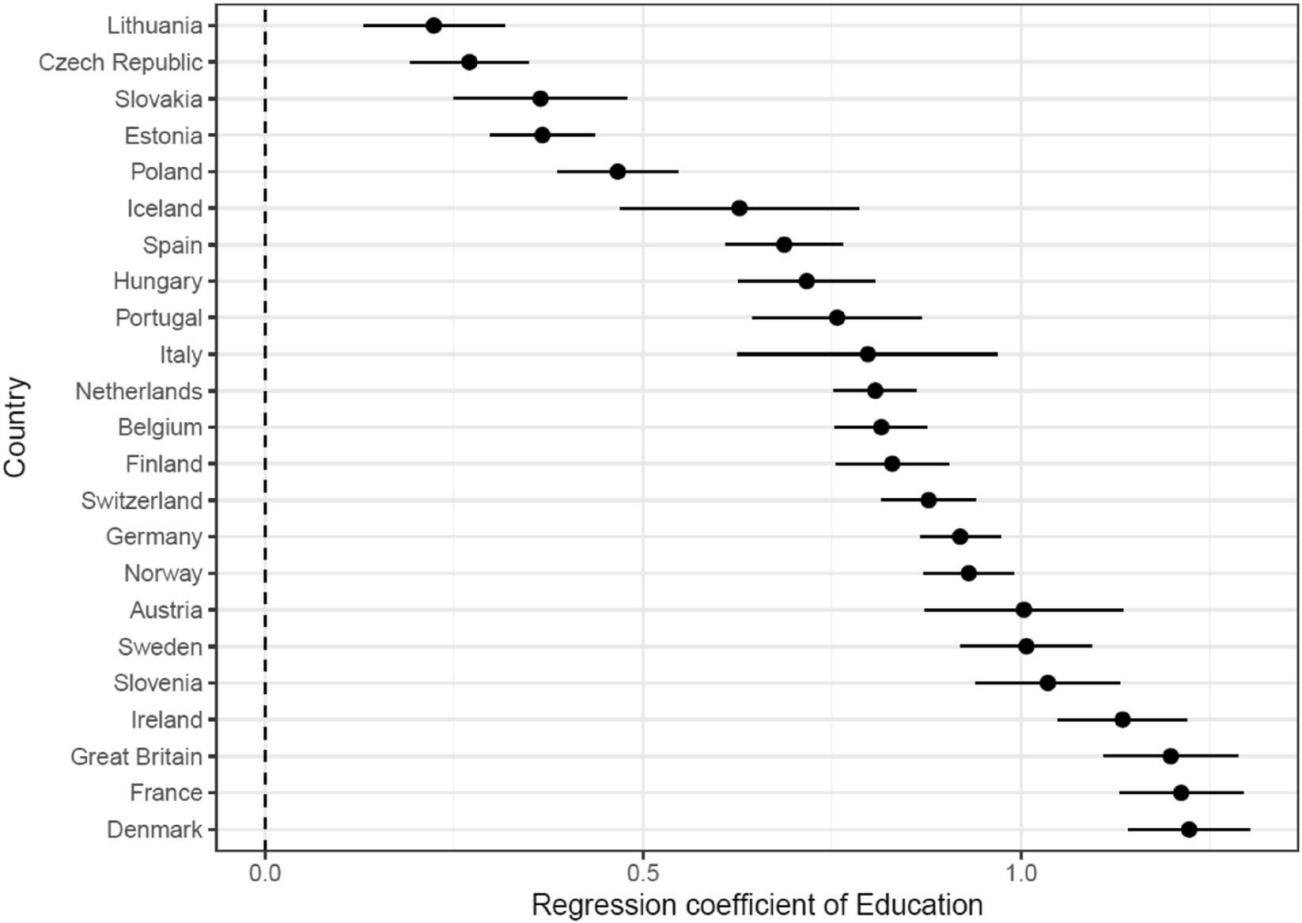
The effects of *education* on *immigration attitudes* per country (regression coefficients and 95% CIs)

To get a first impression of the education divide over immigration, I regressed, for every country separately, *immigration attitudes* on *education* and the individual‐level control variables. I thereby also included year‐fixed effects (see Figure 1 and Section C in Supporting Information S1 for the full analyses). The regression coefficients of *education* can be interpreted as measures of the magnitude of the education divide because they represent the average difference between lower/medium and higher educated individuals regarding their immigration attitudes. The results show that in all countries under analysis there is a significant education divide over immigration with the higher educated being more positive about immigration and the lower and medium educated being more negative. As expected, there are strong differences between countries (ranging from *b* = 0.22 in Lithuania to *b* = 1.22 in Denmark).^4^

What explains these differences? My main aggregate‐level variables are *social spending* (in percentage of GDP), the *unemployment rate* (an internationally comparable measure of the percentage of unemployed people compared to the labor force), *GDP* per capita, and the *Gini coefficient* (which measures socioeconomic inequality). I also included various controls at the aggregate level. In line with previous studies I included, for instance, the level of *liberal democracy*, *trade union density*, and *religious fractionalization* (see Coenders & Scheepers, 2003; Finseraas, 2019; Hello et al., 2002). Details can be found in Sections A and B in Supporting Information S1. All aggregate‐level variables are lagged 1 year.

The main analyses consist of multilevel models with individuals (level 1) nested in country‐years (level 2), nested in countries (level 3). I included year‐fixed effects as well as robust standard errors. Next to random intercepts at the country‐year level, I also estimated random slopes for *education*. I assume, after all, that the effects of *education* on *immigration attitudes* will differ between country‐years. The observations are weighted using the population size weights and the design weights provided by the ESS. My main interest is in the interactions between the aggregate‐level socioeconomic variables and education, because I expect that the effect of *education* on *immigration attitudes* (the education divide over immigration) is conditional upon socioeconomic context. To control for moderation by the other aggregate‐level control variables I also interacted education with all other contextual variables. To ease interpretation, below I present interaction plots vis‐à‐vis the aggregate‐level socioeconomic variables and *education*. The full regression tables can be found in Section E in Supporting Information S1.

## 5 FINDINGS

**FIGURE 2:**
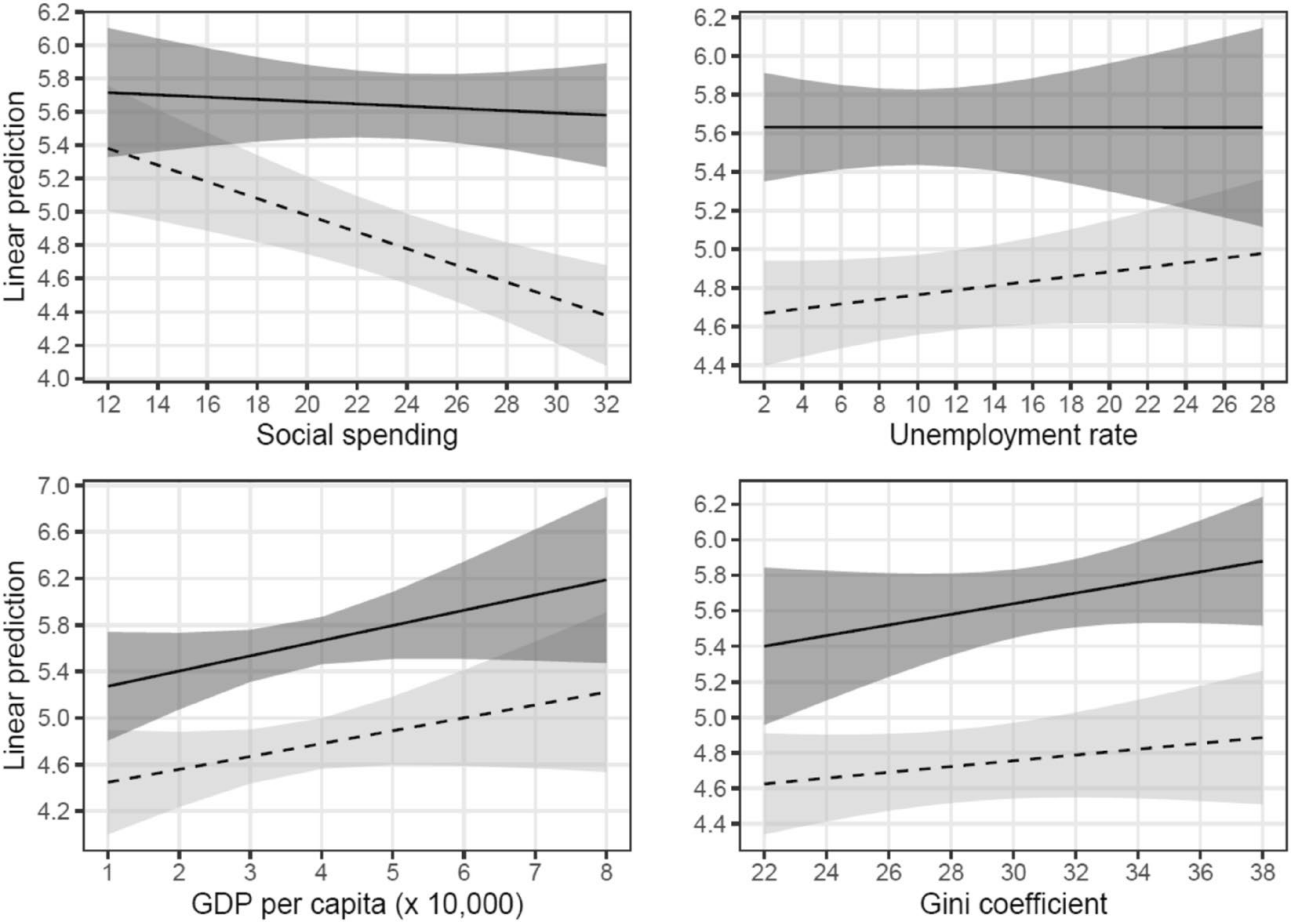
Predictive margins of *education* (DV = *immigration attitudes*) conditional on several socioeconomic indicators (dashed line = lower and medium educated, solid line = higher educated, including 95% CIs)

The effects of *education* on *immigration attitudes* conditional on economic context are summarized in Figure 2. The upper left panel shows that the effect of *education* is strongly conditional on the degree of *social spending*. When countries spend more on welfare, the diversity divide is wider. The other panels demonstrate, however, that there is no such effect when it comes to the *unemployment rate*, *GDP* per capita and the *Gini coefficient*.^5^ Apparently, it is not so much the extent to which an economy flourishes that explains how wide the education gap over immigration is, but the extent to which the state protects its more vulnerable citizens. This is in line with my expectation that the divide can be expected to be wider when individuals live under conditions in which there is a more extensive economic safety net.^6^

The upper left panel also indicates that the found effect of *social spending* on the education gap is due to the susceptibility of the lower and medium educated. The panel shows that *social spending* only exerts an effect on *immigration attitudes* among the lower and medium educated. In other words, in countries in which the state spends more money on welfare, only the lower and medium educated are more strongly anti‐immigration—the effect is not present among the higher educated.

I executed several robustness checks to assess the sensitivity of these results: (1) I included additional or different controls (Sections F and G in Supporting Information S1); (2) I operationalized my main dependent and independent variables differently (Sections I and J in Supporting Information S1); and (3) I relied on alternative model specifications (Sections H and K in Supporting Information S1). These analyses show that my findings are strongly robust to these alternative operationalization and modeling strategies. In all models the interaction between *education* and *social spending* is statistically significant.

Interestingly, these additional analyses also indicate that similar patterns can be observed when the interaction with class instead of education is examined. I will get back to that finding in the concluding section below.

## 6 CONCLUSION

There exists a European‐wide education divide over immigration: in every European country higher educated citizens are more cosmopolitan than their less well‐educated counterparts (Bovens & Wille, 2017; Kriesi et al., 2006, 2008). Yet the width of this education gap strongly differs between countries (Coenders & Scheepers, 2003; Finseraas, 2019; Hello et al., 2002; Weil, 1985). What explains these differences? I have demonstrated that welfare generosity plays an important role. The education gap is particularly pronounced when welfare spending is high, because under such circumstances lower and medium educated citizens are more negative about immigration than when the levels of social spending are lower.

It is important to emphasize that similar patterns can be observed when examining the conditional effects of class instead of education. This suggests that the main argument could be extended beyond the education divide. Although education is probably the main variable distinguishing the “winners” from the “losers” of globalization, it is not the only one. Future studies might want to examine in more detail the interactions with other socio‐economic variables.

The findings reported here have important implications. That the education gap is wider in more generous countries implies that welfare state generosity can also have unintended consequences. Specifically, economic security can fuel cultural clashes between different socioeconomic groups (Inglehart, 1977), and, hence, fuel social polarization. This, of course, does not mean that welfare generosity is a bad idea. Yet it does suggest that we should more carefully think about the ways in which the reduction of socioeconomic hardship can fuel conflicts. Future studies might well want to focus on the concrete mechanisms underlying these processes, and thereby examine how these negative consequences could be mitigated.

## Supporting information

Supplementary Material 1

## Data Availability

The data that support the findings of this study are openly available in Open Science Framework at http://doi.org/10.17605/OSF.IO/KGWSP.

## ENDNOTES

^1^

https://www.economist.com/briefing/2016/07/30/drawbridges‐up.

^2^
This is not to say that the issue of immigration is a *purely* cultural issue. Immigration also has, for instance, important economic and ethical components. However, previous studies suggest that attitudes about immigration are strongly determined by cultural factors. Van der Brug and Van Spanje (2009), for instance, show that public opinion is to a large extent structured by two dimensions: a socio‐economic dimension and a socio‐cultural dimension. The socio‐cultural dimension structures, most importantly, positions on immigration.

^3^
Although it would have been interesting to make a distinction between different categories of university schooled respondents (e.g., more vs. less technical studies), this was, unfortunately, not possible with the existing comparative ES‐ISCED measure provided by the ESS.

^4^
To take into account cross‐country variation in the distribution of the dependent variable, I have also estimated models in which the dependent variable is standardized within countries. This leads to highly similar results. See Section D in Supporting Information S1.

^5^
If we include the interactions separately (and, hence, do not control for the other moderators), also the effect of *GDP* per capita becomes significant in the expected direction. See Section H in Supporting Information S1.

^6^
In line with previous studies, the strength of *liberal democracy* also affects the education divide, see Section E in Supporting Information S1.
