# Supplementary material for: The education gap over immigration and socioeconomic security
Source: Br J Sociol. 2022 Jun 25;73(4):699–705. doi: 10.1111/1468-4446.12959 (PMC9544512; doi:10.1111/1468-4446.12959)
Supplement: Supplementary file 1 — Supplementary Material 1 [file BJOS-73-699-s001.pdf]

Supportive Information: "**The education gap over immigration and socioeconomic security**"

Contents

A: Operationalization of variables 2

B: Descriptive statistics 3

C: Effects of *education* per country 4

D: Effects of *education* per country with standardized dependent variable 7

E: Main analyses 11

F: Analyses with additional/different controls 12

G: Interactions with class/income 14

H: Analyses with interactions separately 16

I: Analyses with alternative operationalizations of *immigration attitudes* 17

J: Analyses with alternative operationalizations of *education* 18

K: Analyses based on alternative modeling strategies 23

References 24

A: Operationalization of variables

To assess individuals' *immigration attitudes*, I constructed a scale which consists of three items. Respondents could answer on a 11-points scale what they thought of the following three questions: (1) "Would you say it is generally bad or good for [respondent's country]'s economy that people come to live here from other countries?" (0 = bad; 10 = good); (2) "Would you say that [country]'s cultural life is generally undermined or enriched by people coming to live here from other countries?" (0 = undermined; 10 = enriched); and (3) "Is [country] made a worse or a better place to live by people coming to live here from other countries?" (0 = worse; 10 = better). I have recoded these items so that "0" refers to an anti-immigration attitude and "10" to a pro-immigration attitude (Cronbach's Alpha = 0.85).

To measure *education* I employed the by the ESS provided ES-ISCED categorization: (I) "less than lower secondary"; (II) "lower secondary"; (IIIb) "upper secondary, vocational, or no access to V1"; (IIIa) "upper secondary, general and/or access to V1"; (IV) "advanced vocational, sub-degree"; (V1) "lower tertiary education, BA level"; (V2) "higher tertiary education, MA level or higher". I dichotomized this variable so that one category (0) represents the lower and middle educated (original categories I through IIIa; 65%) and the other category (1) represents the higher educated (original categories IV through V2; 35%).

To make sure that my assessment of the educational divide over immigration is not confounded by other variables, I controlled for various individual-level characteristics: *gender* (1 = female), *age*, whether someone is *unemployed* (1 = yes) and *subjective income*. I included a subjective measure of income, because including actual income generates a large number of missing values. The variable is measured by asking respondents how they feel about their household's income. It is measured on a 4-points scale, ranging from "very difficult on present income" (1) to "living comfortably on present income" (4). I also assessed whether someone is a *member of a trade union* or a similar organization (1 = yes) and whether someone belongs to a *minority ethnic group* (1 = yes). Finally, I included an item assessing a respondent's *religiosity*, ranging from "not at all religious" (0) to "very religious" (10).

The main aggregate-level variables are *social spending* (in percentage of GDP), the harmonized *unemployment rate* (an internationally comparable measure of the percentage of unemployed people compared to the labor force), *GDP per capita*, and the *Gini coefficient* (which measures socioeconomic inequality). The measures of spending, unemployment and GDP all come from the Organisation for Economic Co-operation and Development (OECD). The Gini coefficients are retrieved from Eurostat.

I also included various controls at the aggregate level. First, I included a measure of the strength of *liberal democracy*. It is measured by means of V-Dem's 'electoral democracy index' (Coppedge et al., 2019). Second, I included *trade union density* (data come from the OECD). Third, I controlled for *religious fractionalization*. The data come from a data set compiled by Alesina, Devleeschauwer, Easterly, Kurlat, and Wacziarg (2003). Fourth, I included a variable measuring the percentage of the 25-64 year-old population that has completed *tertiary education* (retrieved from the OECD). Fifth, because attitudes about immigration might well be affected by the presence of populist radical right parties, I also included a variable measuring the percentage of votes for this party family (*votes radical right*). The information comes from ParlGov (Döring & Manow, 2021) and the categorization is based on The PopuList (Rooduijn et al., 2019). Finally, I controlled for the percentage of *refugees* in a country. The data come from the Worldbank.

B: Descriptive statistics

Variable	Obs	Mean	Std. Dev.	Min	Max
immigration attitudes	209090	5.20	2.10	0.00	10.00
education	209090	0.35	0.48	0.00	1.00
gender	209090	0.52	0.50	0.00	1.00
age	209090	49.35	17.71	18.00	104.00
unemployed	209090	0.05	0.21	0.00	1.00
subjective income (ref. = 1)					
2	209090	0.16	0.36	0.00	1.00
3	209090	0.46	0.50	0.00	1.00
4	209090	0.33	0.47	0.00	1.00
member of trade union	209090	0.20	0.40	0.00	1.00
minority ethnic group	209090	0.05	0.22	0.00	1.00
religiosity	209090	4.36	3.05	0.00	10.00
social spending	209090	22.85	4.60	12.57	31.98
unemployment rate	209090	8.23	3.88	2.56	26.12
GDP per capita	209090	37709.93	12090.25	11101.11	77679.03
Gini coefficient	209090	28.50	3.45	22.00	37.90
liberal democracy	209090	0.88	0.04	0.62	0.93
union density	209090	29.23	20.44	4.30	90.00
religious fractionalization	209090	0.43	0.19	0.14	0.72
% tertiary education	209090	30.64	8.22	11.13	45.74
% votes radical right	209090	7.31	11.11	0.00	70.40
% refugees	209090	0.36	0.41	0.00	2.40

C: Effects of *education* per country

	AT	BE	CH	CZ	DE	DK	EE	ES
education	2.729***	2.260***	2.406***	1.310***	2.508***	3.395***	1.443***	1.987***
gender	1.171**	0.830***	0.953	1.091**	0.975	1.004	1.042	0.781***
age	0.994**	0.989***	0.995***	0.986***	0.989***	0.985***	0.980***	0.990***
unemployed	0.974	0.928	1.086	0.899	0.770***	1.013	0.837	0.820**
subjective income (ref. = 1)								
2	2.031***	1.543***	1.287*	1.622***	1.568***	0.942	1.07	1.403***
3	2.996***	2.095***	1.269*	2.234***	2.274***	1.055	1.573***	1.608***
4	4.624***	2.807***	1.504***	2.800***	3.456***	1.369	2.378***	2.079***
member of trade union	0.943	0.888***	1.261***	0.879	1.254***	0.983	1.076	1.237***
minority ethnic group	4.144***	2.282***	1.500***	1.556***	1.892***	2.041***	1.318***	2.123***
religiosity	1.006	1.043***	1.014**	1.067***	1.052***	1.023**	1.053***	0.973***
constant	28.331***	73.253***	222.175***	62.569***	95.911***	218.791***	95.749***	212.822***
N	5601	14670	13825	13821	23004	9474	12893	12830

* p<0.05, ** p<0.01, *** p<0.001, year dummies included

	FI	FR	GB	HU	IE	IS	IT	LT
education	2.292***	3.360***	3.314***	2.047***	3.109***	1.873***	2.220***	1.250***
gender	1.200***	0.889**	0.801***	0.861***	0.809***	1.229*	1.124	1.112*
age	0.996***	0.984***	0.982***	0.993***	0.993***	0.995*	0.987***	0.985***
unemployed	0.912	1.072	0.907	0.853	0.811**	0.833	0.83	0.905
subjective income (ref. = 1)	1	1	1	1	1	1	1	1
2	1.281*	0.991	1.301*	1.474***	1.866***	1.078	1.702***	1.199*
3	1.700***	1.24	2.004***	1.802***	2.853***	1.146	2.485***	1.589***
4	2.552***	1.865***	3.075***	2.113***	3.875***	1.357	4.439***	2.221***
member of trade union	0.917*	1.584***	1.023	1.067	0.879*	0.92	1.443**	0.928
minority ethnic group	1.967***	2.472***	2.158***	1.028	3.036***	0.982	9.176***	0.904
religiosity	1.024***	1.007	1.071***	1.028***	0.984*	0.965*	0.981	1.064***
constant	139.687***	127.288***	63.245***	59.944***	58.896***	537.777***	101.087***	118.873***
N	9220	12812	9939	11603	11376	1856	5352	7345

* $p<0.05$, ** $p<0.01$, *** $p<0.001$, year dummies included

	NL	NO	PL	PT	SE	SI	SK
education	2.242***	2.537***	1.594***	2.132***	2.738***	2.816***	1.439***
gender	1.008	1.126***	0.929*	0.836***	1.243***	0.939	0.954
age	0.996***	0.994***	0.993***	0.991***	0.987***	0.992***	0.986***
unemployed	1.134	1.051	0.860*	0.941	1.071	1.047	0.886
subjective income (ref. = 1)							
2	1.296**	1.453**	1.811***	1.431***	1.635**	1.344*	1.227**
3	1.659***	1.782***	2.594***	1.961***	1.934***	1.934***	1.830***
4	2.084***	2.170***	3.426***	2.977***	2.729***	3.103***	2.205***
member of trade union	1.214***	1.285***	1.231**	1.054	1.035	1.025	1.204*
minority ethnic group	2.361***	1.853***	1.263	3.000***	1.594***	2.130***	1.199
religiosity	1.015***	1	0.954***	1.009	1.039***	0.994	1.027***
constant	80.526***	89.103***	164.046***	128.366***	296.878***	62.132***	103.620***
N	15399	13590	12165	8556	7439	10068	7719

* $p<0.05$, ** $p<0.01$, *** $p<0.001$, year dummies included

D: Effects of *education* per country with standardized dependent variable

	AT	BE	CH	CZ	DE	DK	EE	ES
education	1.569***	1.558***	1.648***	1.149***	1.576***	1.833***	1.202***	1.407***
gender	1.071**	0.903***	0.973	1.047**	0.988	1.001	1.021	0.883***
age	0.998**	0.994***	0.997***	0.993***	0.994***	0.992***	0.990***	0.995***
unemployed	0.989	0.958	1.049	0.949	0.875***	1.008	0.912*	0.909**
subjective income (ref. = 1)								
2	1.370***	1.265***	1.158*	1.276***	1.249***	0.976	1.034	1.190***
3	1.630***	1.493***	1.147*	1.502***	1.499***	1.03	1.254***	1.275***
4	1.977***	1.751***	1.265***	1.687***	1.843***	1.172	1.541***	1.450***
member of trade union	0.972	0.938***	1.142***	0.936	1.120***	0.993	1.037	1.114***
minority ethnic group	1.884***	1.565***	1.263***	1.252***	1.371***	1.424***	1.154***	1.474***
religiosity	1.003	1.023***	1.008**	1.033***	1.025***	1.011**	1.026***	0.987***
constant	0.558***	0.719***	0.774***	0.892*	0.663***	1.015	1.139**	0.996
N	5601	14670	13825	13821	23004	9474	12893	12830

* $p<0.05$, ** $p<0.01$, *** $p<0.001$, year dummies included

	FI	FR	GB	HU	IE	IS	IT	LT
education	1.585***	1.740***	1.696***	1.432***	1.647***	1.439***	1.391***	1.116***
gender	1.105***	0.948**	0.907***	0.929***	0.912***	1.127*	1.049	1.054*
age	0.998***	0.993***	0.992***	0.997***	0.997***	0.997*	0.994***	0.992***
unemployed	0.95	1.03	0.958	0.922	0.912**	0.896	0.926	0.943
subjective income (ref. = 1)								
2	1.148*	0.996	1.122*	1.216***	1.307***	1.043	1.245***	1.095*
3	1.346***	1.102	1.357***	1.347***	1.576***	1.08	1.455***	1.253***
4	1.685***	1.327***	1.639***	1.461***	1.808***	1.192	1.848***	1.484***
member of trade union	0.954*	1.235***	1.011	1.031	0.945*	0.953	1.164**	0.971
minority ethnic group	1.450***	1.512***	1.403***	1.013	1.632***	0.989	2.497***	0.955
religiosity	1.013***	1.003	1.031***	1.014***	0.992*	0.980*	0.992	1.031***
constant	0.617***	1.045	0.805***	0.827***	0.694***	0.924	0.839*	1.038
N	9220	12812	9939	11603	11376	1856	5352	7345

* $p<0.05$, ** $p<0.01$, *** $p<0.001$, year dummies included

	NL	NO	PL	PT	SE	SI	SK
education	1.653***	1.677***	1.283***	1.480***	1.676***	1.648***	1.214***
gender	1.006	1.068***	0.961*	0.908***	1.117***	0.967	0.974
age	0.998***	0.996***	0.996***	0.996***	0.993***	0.996***	0.993***
unemployed	1.079	1.028	0.922*	0.967	1.033	1.016	0.931
subjective income (ref. = 1)							
2	1.173**	1.234**	1.380***	1.213***	1.290**	1.147*	1.108*
3	1.367***	1.382***	1.675***	1.427***	1.405***	1.378***	1.366***
4	1.576***	1.541***	1.939***	1.773***	1.674***	1.731***	1.503***
member of trade union	1.127***	1.150***	1.112**	1.034	1.018	1.011	1.098*
minority ethnic group	1.708***	1.408***	1.119	1.795***	1.269***	1.439***	1.106*
religiosity	1.009***	1	0.976***	1.004	1.020***	0.997	1.014***
constant	0.617***	0.618***	0.867*	0.92	0.640***	0.777***	1.023
N	15399	13590	12165	8556	7439	10068	7719

* $p<0.05$, ** $p<0.01$, *** $p<0.001$, year dummies included

Figure 1. The effects of *education* on *immigration attitudes* per country, with standardized dependent variable (regression coefficients and 95% CIs)

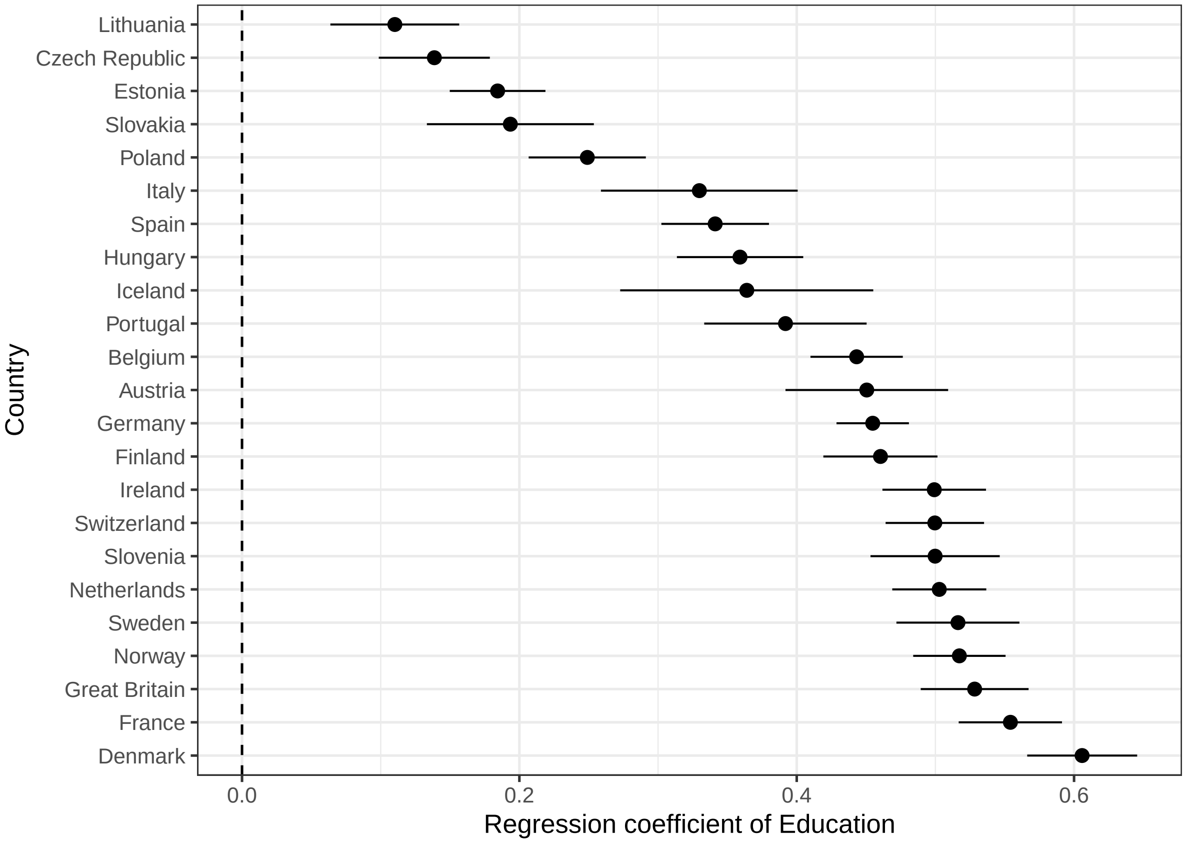

E: Main analyses

	M1	M2	M3
education	0.807***	0.804***	-2.597*
gender	-0.087*	-0.087*	-0.087*
age	-0.012***	-0.012***	-0.012***
unemployed	-0.130**	-0.130**	-0.130**
subjective income (ref. = 1)			
2	0.373***	0.373***	0.373***
3	0.683***	0.683***	0.685***
4	1.043***	1.043***	1.044***
member of trade union	0.160***	0.160***	0.159***
minority ethnic group	0.769***	0.768***	0.769***
religiosity	0.020*	0.020*	0.020*
social spending		-0.043**	-0.050***
unemployment rate		0.009	0.012
GDP per capita		0.000	0.000
Gini coefficient		0.018	0.016
liberal democracy		-1.633	-1.79
union density		0.016***	0.016***
religious fractionalization		-0.256	-0.318
% tertiary education		0.019*	0.018
% votes radical right		-0.015***	-0.015***
% refugees		-0.177	-0.179
education * social spending			0.043***
education * unemployment rate			-0.012
education * GDP per capita			0.000
education * Gini coefficient			0.014
education * liberal democracy			2.003**
education * union density			0.001
education * religious fractionalization			0.282
education * % tertiairy education			0.004
education * % votes radical right			0.004
education * % refugees			-0.01
constant	4.624***	5.414***	5.762***
N	209090	209090	209090

* p<0.05, ** p<0.01, *** p<0.001, year dummies included

F: Analyses with additional/different controls

- M1: class (6 categories, based on the Erikson-Goldthorpe-Portocarero (EGP) classification scheme) and income (in deciles) as additional individual-level controls
- M2: instead of % refugees I included % foreign born (stock of foreign-born population in OECD countries, that is, the size of immigrant populations as measured in number of persons relative to the total population size [retrieved from OECD])
- M3: instead of % refugees I included % migrant stock (international migrant stock is the number of people born in a country other than that in which they live, relative to the total population size [retrieved from World Bank])
- M4: instead of % refugees I included % permanent immigration inflows (standardized measure of the regulated movements of foreigners considered to be settling in the country from the perspective of the destination country, covering regulated movements of foreigners as well as free movement migration, relative to the total population size [retrieved from OECD])
- M5: instead of % refugees I included % non-Western people. In the ESS I computed, per country-year, the percentage of respondents for whom their father was not born in Europe, Australia or North America (Gorodzeisky & Semyonov, 2018).

	M1	M2	M3	M4	M5
education	-3.800***	-3.364**	-1.749	-4.740***	-2.800*
gender	-0.128**	-0.092*	-0.087*	-0.097*	-0.089*
age	-0.009***	-0.012***	-0.012***	-0.012***	-0.012***
unemployed	-0.094	-0.111*	-0.130**	-0.131*	-0.121**
subjective income (ref. = 1)					0
2	0.350***	0.383***	0.373***	0.380***	0.357***
3	0.578***	0.695***	0.685***	0.676***	0.678***
4	0.830***	1.062***	1.044***	1.040***	1.045***
class	0.139***				
income	0.022***				
member of trade union	0.151***	0.162***	0.160***	0.171***	0.160***
minority ethnic group	0.775***	0.773***	0.769***	0.809***	0.765***
religiosity	0.019*	0.021*	0.020*	0.024*	0.021*
social spending	-0.055***	-0.060***	-0.058***	-0.068***	-0.052***
unemployment rate	0.003	0.006	0.011	-0.007	0.015
GDP per capita	0	0	0	0	0.000*
Gini coefficient	0.014	0.006	0.02	0.001	0.022
liberal democracy	-1.859	-2.612*	-1.749	-5.027*	-1.762
union density	0.015**	0.014*	0.015***	0.006	0.014**
religious fractionalization	-0.578	-0.474	-0.523	-0.586	-0.503
% tertiary education	0.014	0.012	0.01	0.036**	0.009
% votes radical right	-0.013***	-0.017***	-0.015***	-0.002	-0.015***
% refugees	-0.249*				
% foreign born		0.011			
% migrant stock			0.012		
% immigration inflows				-0.177	
% non-Western					-0.005
education * social spending	0.046***	0.049***	0.042***	0.032**	0.044***
education * unemployment rate	-0.001	-0.015	-0.014	-0.018*	-0.013
education * GDP per capita	0	0	0	0	0
education * Gini coefficient	0.011	0.015	0.008	0.039*	0.019
education * liberal democracy	2.773***	2.792***	1.359	4.086***	1.968*
education * union density	-0.001	0.001	0.001	0.001	0.001
education * religious fractionalization	0.377	0.592*	0.183	0.158	0.271
education * % tertiairy education	0.01	0.001	0.003	0.004	0.004
education * % votes radical right	0.002	0.007*	0.002	0.005	0.004
education * % refugees	0.035				
education * % foreign born		0.003			
education * % migrant			0.018		
education * % immigration inflows				-0.188	
education * % non-Western					0.002
constant	0.646***	0.676***	0.668***	0.667***	5.578***
N	1.12E+05	1.76E+05	2.09E+05	1.25E+05	1.95E+05

G: Interactions with class/income

- M1: interactions with class (6 categories, based on the Erikson-Goldthorpe-Portocarero (EGP) classification scheme) instead of education
- M2: interactions with income (in deciles) instead of education
- M3: interactions of education with social spending and liberal democracy, of class with social spending and liberal democracy, and of income with social spending and liberal democracy (and random slopes for education, class and income)

	M1	M2	M3
class	-0.884***	0.134***	-0.234*
income	0.023***	-0.549***	-0.048
education	0.727***	0.729***	-1.928*
gender	-0.130**	-0.128**	-0.133**
age	-0.010***	-0.009***	-0.009***
unemployed	-0.094	-0.08	-0.089
subjective income (ref. = 1)			0
2	0.347***	0.347***	0.349***
3	0.573***	0.562***	0.578***
4	0.825***	0.823***	0.832***
member of trade union	0.153***	0.155***	0.153***
minority ethnic group	0.787***	0.785***	0.774***
religiosity	0.019*	0.020**	0.019*
social spending	-0.075***	-0.067***	-0.074***
unemployment rate	0.003	0.009	0.002
GDP per capita	0	0	0
Gini coefficient	0.001	-0.015	0
liberal democracy	-2.597	-2.719	-2.576
union density	0.011*	0.012*	0.011*
religious fractionalization	-0.922	-0.563	-0.692
% tertiary education	0.018*	0.024*	0.020**
% votes radical right	-0.015***	-0.015***	-0.015***
% refugees	-0.275**	-0.101	-0.222*
class * social spending	0.010***		0.005**
class * unemployment rate	0		
class * GDP per capita	0		
class * Gini coefficient	0.005		
class * liberal democracy	0.539***		0.289*
class * union density	0.001*		
class * religious fractionalization	0.148**		
class * % tertiairy education	0		
class * % votes radical right	0.001		
class * % refugees	0.011		
income * social spending		0.005***	0.001
income * unemployment rate		-0.001	
income * GDP per capita		0	
income * Gini coefficient		0.007**	
income * liberal democracy		0.305**	0.034
income * union density		0.001	
income * religious fractionalization		0.034	
income * % tertiairy education		-0.002	
income * % votes radical right		0	
income * % refugees		-0.022*	
education * social spending			0.028*
education * liberal democracy			2.180*
constant	7.699***	7.630***	7.476***
N	1.12E+05	1.12E+05	1.12E+05

H: Analyses with interactions separately

	M1	M2	M3	M4
education	-0.117	0.905***	0.456**	1.217*
gender	-0.087*	-0.087*	-0.087*	-0.087*
age	-0.012***	-0.012***	-0.012***	-0.012***
unemployed	-0.130**	-0.130**	-0.130**	-0.130**
subjective income (ref. = 1)				
2	0.373***	0.373***	0.373***	0.373***
3	0.683***	0.684***	0.684***	0.684***
4	1.043***	1.044***	1.044***	1.043***
member of trade union	0.160***	0.160***	0.159***	0.160***
minority ethnic group	0.769***	0.768***	0.768***	0.769***
religiosity	0.020*	0.020*	0.020*	0.020*
social spending	-0.049***	-0.042**	-0.042**	-0.043**
unemployment rate	0.010	0.010	0.009	0.009
GDP per capita	0.000	0.000	0.000	0.000
Gini coefficient	0.018	0.018	0.018	0.019
liberal democracy	-1.597	-1.636	-1.596	-1.621
union density	0.016***	0.016***	0.016***	0.016***
religious fractionalization	-0.28	-0.26	-0.235	-0.257
% tertiary education	0.019*	0.019*	0.019	0.019*
% votes radical right	-0.015***	-0.015***	-0.015***	-0.015***
% refugees	-0.178	-0.177	-0.176	-0.177
education * social spending	0.040***			
education * unemployment rate		-0.012		
education * GDP per capita			0.000**	
education * Gini coefficient				-0.014
Constant	5.489***	5.417***	5.402***	5.372***
N	209090	209090	209090	209090

* p<0.05, ** p<0.01, *** p<0.001, year dummies included

I: Analyses with alternative operationalizations of *immigration attitudes*

- M1: immigration and economy ("Would you say it is generally bad or good for [respondent's country]'s economy that people come to live here from other countries?")
- M2: immigration and culture ("Would you say that [country]'s cultural life is generally undermined or enriched by people coming to live here from other countries?")
- M3: immigration general ("Is [country] made a worse or a better place to live by people coming to live here from other countries?")

	M1	M2	M3
education	-2.915**	-3.047**	-2.265
gender	-0.287***	0.069	-0.042
age	-0.007***	-0.014***	-0.014***
unemployed	-0.171***	-0.104*	-0.114*
subjective income (ref. = 1)	0.000	0.000	0.000
2	0.387***	0.361***	0.373***
3	0.744***	0.651***	0.661***
4	1.165***	0.994***	0.974***
member of trade union	0.130*	0.194***	0.155***
minority ethnic group	0.707***	0.759***	0.840***
religiosity	0.021*	0.007	0.032***
social spending	-0.062***	-0.033	-0.056***
unemployment rate	-0.012	0.024*	0.024**
GDP per capita	0.000	0.000	0.000
Gini coefficient	0.028	0.021	0.003
liberal democracy	-2.250*	-1.654	-1.39
union density	0.010*	0.018***	0.018***
religious fractionalization	-0.439	-0.232	-0.355
% tertiary education	0.01	0.025*	0.021
% votes radical right	-0.015***	-0.019***	-0.011***
% refugees	-0.181	-0.172	-0.182
education * social spending	0.046***	0.048***	0.039***
education * unemployment rate	-0.005	-0.015	-0.016*
education * GDP per capita	0.000	0.000	0.000
education * Gini coefficient	0.011	0.008	0.027
education * liberal democracy	2.373***	2.752***	1.106
education * union density	0.000	-0.001	0.004
education * religious fractionalization	0.329	0.188	0.399
education * % tertiairy education	0.003	0.005	0.004
education * % votes radical right	0.005	0.005	0.001
education * % refugees	-0.041	-0.032	0.032
Constant	6.184***	5.495***	5.432***
N	209090	209090	209090

* $p<0.05$, ** $p<0.01$, *** $p<0.001$, year dummies included

J: Analyses with alternative operationalizations of *education*

- M1: Education in years.
- M2: Education measured with the original ES-ISCED categorization provided by the ESS (i.e., 7 categories, see section A), as a *continuous* variable.
- M3: Education measured with the original ES-ISCED categorization, but as a *categorical* variable.
- M4: Education measured with 3 categories: low (ES-ISCED I-II), middle (ES-ISCED III), and high (ES-ISCED IV-V).

	M1	M2	M3	M4
gender	-0.077*	-0.077*	-0.088*	-0.081*
age	-0.006***	-0.008***	-0.008***	-0.010***
unemployed	-0.124**	-0.113*	-0.110*	-0.117**
subjective income (ref. = 1)	0	0	0	0
2	0.317***	0.322***	0.324***	0.336***
3	0.604***	0.588***	0.585***	0.619***
4	0.952***	0.872***	0.854***	0.960***
member of trade union	0.141***	0.124**	0.136**	0.139**
minority ethnic group	0.824***	0.785***	0.754***	0.794***
religiosity	0.024**	0.024**	0.023**	0.022*
social spending	-0.094***	-0.085***	-0.070***	-0.058***
unemployment rate	0.016	0.008	0.008	-0.001
GDP per capita	0	0	0	0
Gini coefficient	-0.006	0.004	0.006	0.001
liberal democracy	-3.672*	-3.034*	-1.112	-0.382
union density	0.012*	0.015**	0.016***	0.012**
religious fractionalization	-1.265	-0.679	-0.433	-0.559
% tertiary education	0.011	0.023*	0.036***	0.019
% votes radical right	-0.016**	-0.017***	-0.004	-0.010***
% refugees	-0.2	-0.122	0.049	0.055
education	-0.361*	-0.705*		
education (ref. = ES-ICED I)				
ES-ISCED II			-0.98	
ES-ISCED IIIb			2.107	
ES-ISCED IIIa			-0.152	
ES-ISCED IV			-1.573	
ES-ISCED V1			-2.888	
ES-ISCED V2			-4.193*	
education (ref. = low)				
middle				1.224
high				-2.808*

	M1	M2	M3	M4
education * social spending	0.005***	0.013***		
education * unemployment rate	-0.002**	-0.003		
education * GDP per capita	0	0		
education * Gini coefficient	0.002	0.004		
education * liberal democracy	0.307**	0.577**		
education * union density	0	0		
education * religious fractionalization	0.051	0.091		
education * % tertiairy education	0	0		
education * % votes radical right	0	0.001		
education * % refugees	0.008	0.011		
education * social spending (ref. = ES-ICED I)				
ES-ISCED II			0.030**	
ES-ISCED IIIb			-0.003	
ES-ISCED IIIa			0.036***	
ES-ISCED IV			0.060***	
ES-ISCED V1			0.068***	
ES-ISCED V2			0.072***	
education * unemployment rate (ref. = ES-ICED I)				
ES-ISCED II			-0.018	
ES-ISCED IIIb			0.015	
ES-ISCED IIIa			-0.004	
ES-ISCED IV			-0.033*	
ES-ISCED V1			-0.034*	
ES-ISCED V2			-0.011	
education * GDP per capita (ref. = ES-ICED I)				
ES-ISCED II			0	
ES-ISCED IIIb			0.000***	
ES-ISCED IIIa			0.000**	
ES-ISCED IV			0	
ES-ISCED V1			0	
ES-ISCED V2			0	
education * Gini coefficient (ref. = ES-ICED I)				
ES-ISCED II			0.005	
ES-ISCED IIIb			0.004	
ES-ISCED IIIa			0.015	
ES-ISCED IV			0.008	
ES-ISCED V1			0.02	
ES-ISCED V2			0.027	

	M1	M2	M3	M4
education * liberal democracy (ref. = ES-ICED I)				
ES-ISCED II			0.607	
ES-ISCED IIIb			-2.337	
ES-ISCED IIIa			-0.411	
ES-ISCED IV			1.675	
ES-ISCED V1			1.939	
ES-ISCED V2			2.921*	
education * union density (ref. = ES-ICED I)				
ES-ISCED II			-0.003	
ES-ISCED IIIb			0.002	
ES-ISCED IIIa			-0.001	
ES-ISCED IV			-0.005	
ES-ISCED V1			0	
ES-ISCED V2			0.005	
education * religious fractionalization (ref. = ES-ICED I)				
ES-ISCED II			-0.085	
ES-ISCED IIIb			-0.191	
ES-ISCED IIIa			0.438	
ES-ISCED IV			-0.317	
ES-ISCED V1			0.326	
ES-ISCED V2			0.619	
education * % tertiary education (ref. = ES-ICED I)				
ES-ISCED II			-0.004	
ES-ISCED IIIb			-0.017***	
ES-ISCED IIIa			-0.024***	
ES-ISCED IV			-0.013	
ES-ISCED V1			0.009	
ES-ISCED V2			-0.004	
education * % votes radical right (ref. = ES-ICED I)				
ES-ISCED II			-0.007	
ES-ISCED IIIb			-0.021***	
ES-ISCED IIIa			-0.016**	
ES-ISCED IV			-0.005	
ES-ISCED V1			-0.011	
ES-ISCED V2			-0.004	
education * % refugees (ref. = ES-ICED I)				
ES-ISCED II			-0.251*	
ES-ISCED IIIb			-0.147	
ES-ISCED IIIa			-0.025	
ES-ISCED IV			-0.144	
ES-ISCED V1			-0.209	
ES-ISCED V2			-0.149	

	M1	M2	M3	M4
education * social spending (ref. = low)				
middle				0.006
high				0.053***
education * unemployment rate (ref. = low)				
middle				0.012
high				-0.013
education * GDP per capita (ref. = low)				
middle				0.000**
high				0
education * Gini coefficient (ref. = low)				
middle				0.017
high				0.024
education * liberal democracy (ref. = low)				
middle				-1.877*
high				1.935*
education * union density (ref. = low)				
middle				0.004*
high				0.003
education * religious fractionalization (ref. = low)				
middle				-0.074
high				0.395
education * % tertiary education (ref. = low)				
middle				-0.009
high				-0.003
education * % votes rdaical right (ref. = low)				
middle				-0.012***
high				0
education * % refugees (ref. = low)				
middle				-0.137*
high				-0.083
constant				5.248***
N	2.08E+05	2.09E+05	2.09E+05	2.09E+05

K: Analyses based on alternative modeling strategies

- M1: Analysis based on two instead of three levels (i.e., individuals nested in country-years, including country- and year-fixed effects
- M2: Analysis excluding weights.
- M3: Analysis excluding random intercepts for *education*

	M1	M2	M3
education	-2.597***	-1.432	-3.785***
gender	-0.087***	-0.029	-0.086*
age	-0.012***	-0.011***	-0.012***
unemployed	-0.130**	-0.105***	-0.128**
subjective income (ref. = 1)	0.000	0.000	0.000
2	0.373***	0.353***	0.372***
3	0.685***	0.665***	0.683***
4	1.044***	0.984***	1.043***
member of trade union	0.160***	0.074	0.163***
minority ethnic group	0.769***	0.591***	0.773***
religiosity	0.020***	0.025***	0.020*
social spending	-0.057***	-0.046***	-0.056***
unemployment rate	0.005	0.017	0.018
GDP per capita	0.000	0.000	0.000*
Gini coefficient	0.011	0.012	0.012
liberal democracy	-2.533***	-1.734	-2.021
union density	0.037***	0.017***	0.014**
religious fractionalization	1.35	-0.185	-0.458
% tertiary education	0.044*	0.017	0.017
% votes radical right	-0.016***	-0.015***	-0.016***
% refugees	-0.325**	-0.117	-0.183
education * social spending	0.043***	0.039***	0.052***
education * unemployment rate	-0.011	-0.013	-0.015*
education * GDP per capita	0.000	0.000	0.000
education * Gini coefficient	0.013	0.002	0.025
education * liberal democracy	2.028**	1.344*	2.713***
education * union density	0.001	0.000	0.002
education * religious fractionalization	0.273	0.045	0.494*
education * % tertiairy education	0.004	0.000	0.010
education * % votes radical right	0.004	0.000	0.007*
education * % refugees	-0.01	-0.006	-0.105
Constant	5.175**	5.575***	6.178***
N	209090	209090	209090

* p<0.05, ** p<0.01, *** p<0.001, year dummies included

References

Alesina, A., Devleeschauwer, A., Easterly, W., Kurlat, S., & Wacziarg, R. (2003). Fractionalization. *Journal of Economic growth*, *8*(2), 155–194.

Coppedge, M., Gerring, J., Knutsen, C. H., Lindberg, S. I., Teorell, J., Altman, D., ... others (2019). V-dem codebook v9.

Döring, H., & Manow, P. (2021). Parliaments and governments database (parlgov): Information on parties, elections and cabinets in modern democracies. *Development version.*

Gorodzeisky, A., & Semyonov, M. (2018). Competitive threat and temporal change in anti-immigrant sentiment: insights from a hierarchical age-period-cohort model. *Social Science Research*, *73*, 31–44.

Rooduijn, M., Van Kessel, S., Froio, C., Pirro, A., De Lange, S., Halikiopoulou, D., ... Taggart, P. (2019). The populist: an overview of populist, far right, far left and eurosceptic parties in europe.
